# Investigation of Heater Structures for Thermal Conductivity Measurements of SiO_2_ and Al_2_O_3_ Thin Films Using the 3-Omega Method

**DOI:** 10.3390/nano12111928

**Published:** 2022-06-04

**Authors:** Fabian Kühnel, Christoph Metzke, Jonas Weber, Josef Schätz, Georg S. Duesberg, Günther Benstetter

**Affiliations:** 1 Department of Electrical Engineering and Media Technology, Deggendorf Institute of Technology, Dieter-Görlitz-Platz 1, 94469 Deggendorf, Germany; fabian.kuehnel@th-deg.de (F.K.); christoph.metzke@th-deg.de (C.M.); jonas.weber@th-deg.de (J.W.); 2 Faculty of Electrical Engineering and Information Technology, University of the Bundeswehr Munich, Werner-Heisenberg-Weg 39, 85577 Neubiberg, Germany; georg.duesberg@unibw.de; 3 Department of Electrical Engineering, Helmut Schmidt University/University of the Federal Armed Forces Hamburg, Holstenhofweg 85, 22043 Hamburg, Germany; 4 Department of Applied Physics, University of Barcelona, Martí i Franquès 1, 08028 Barcelona, Spain; 5 Infineon Technologies AG, Wernerwerkstraße 2, 93049 Regensburg, Germany; josef.schaetz@infineon.com; 6 Chair of Electronic Devices, RWTH Aachen University, Otto-Blumenthal-Straße 2, 52074 Aachen, Germany

**Keywords:** 3-Omega method, 3*ω*, heater geometry, heater dimensions, thermal conductivity, silicon dioxide (SiO_2_), aluminum oxide (Al_2_O_3_), annealing process

## Abstract

A well-known method for measuring thermal conductivity is the 3-Omega (3*ω*) method. A prerequisite for it is the deposition of a metal heater on top of the sample surface. The known design rules for the heater geometry, however, are not yet sufficient. In this work, heaters with different lengths and widths within the known restrictions were investigated. The measurements were carried out on SiO_2_ thin films with different film thicknesses as a reference. There was a significant difference between theoretical deposited heater width and real heater width, which could lead to errors of up to 50% for the determined thermal conductivity. Heaters with lengths between 11 and 13 mm and widths of 6.5 µm or more proved to deliver the most trustworthy results. To verify the performance of these newfound heaters, additional investigations on Al_2_O_3_ thin films were carried out, proving our conclusions to be correct and delivering thermal conductivity values of 0.81 Wm^−1^ K^−1^ and 0.93 Wm^−1^ K^−1^ for unannealed and annealed samples, respectively. Furthermore, the effect of annealing on Al_2_O_3_ was studied, revealing a significant shrinking in film thickness of approximately 11% and an increase in thermal conductivity of 15%. The presented results on well-defined geometries will help to produce optimized heater structures for the 3*ω* method.

## 1. Introduction

Measuring the thermal conductivity of thin films is of great importance, as these films are vital components within semiconductor devices and integrated circuits [1,2]. Zhao et al. and others [3,4] alluded that the properties of thin films may vary significantly from those of their bulk material counterparts. Therefore, separate investigation is crucial. To this end, various measurement methods may be deployed. Among them are thermoreflectance imaging [5,6], the time-domain thermoreflectance method (TDTR) [7,8], the laser flash method [9,10], the micro-Raman method [11,12], scanning thermal microscopy (SThM) [13,14], and many more.

The 3*ω* method is one of those methods and is commonly used to measure cross-plane thermal conductivity. It was first introduced by O. Corbino in 1910 to measure the thermal diffusivity of a metal filament in light bulbs [15,16]. In 1987, Birge and Nagel investigated the frequency-dependent specific heat of liquids using an immersed thin planar heater [17]. Cahill et al. used a heater deposited on top of dielectric solids in order to measure thermal conductivity for the first time in 1989 [18]. In 1997, Lee et al. adapted the method for thin film measurements down to a thickness of 20 nm [19]. Two years later, in-plane and cross-plane thermal conductivities of anisotropic materials were measured [20,21]. Since 2006, the 3*ω* method has been adapted to many different samples, such as carbon nanotubes [22], gases [23], and free-standing membranes [24].

In this work, a closer look was taken at the heater structure, which is required to perform 3*ω* measurements. It is commonly known that the heater width has to be much larger than the film thickness to measure true cross-plane thermal conductivity. Furthermore, the heater has to be considered infinitely long. These and other requirements have been defined by others and are briefly explained later. Although these requirements exist, there are two major problems:

- Because of the imprecise specifications of heater geometries, researchers define their structures individually [19,22,25,26,27]. This leads to inaccuracies and a large spread of measurement results.
- Even if all requirements are fulfilled, choosing different heater geometries within the boundaries still leads to differing results.

To fix these problems, a comprehensive study of a wide range of heater geometries was performed. The goal thereof was to provide recommendations on valid heater geometries, which should simplify the choice of the right 3*ω* layouts for other researchers. Therefore, first, measurements with different heater structures were performed on well-known SiO_2_ thin films. In a second step, the best heater geometries were tested on Al_2_O_3_ thin films as a verification process.

## 2. Materials and Methods

### 2.1. Three-Omega Method

**Figure 1:**
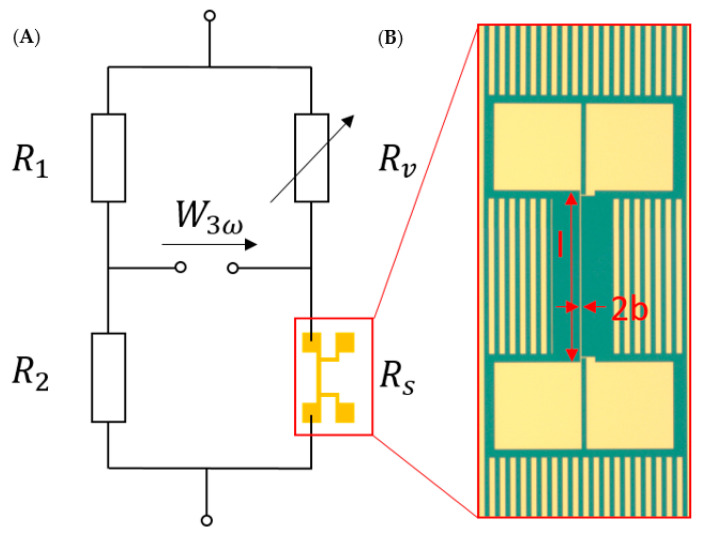
(**A**) Wheatstone bridge layout. R_1_ and R_2_ are high-precision resistances chosen in such a way that most of the current passes through the heater, R_v_ is a variable decade resistance, and R_s_ is the sample resistance. (**B**) 3*ω* metal heater deposited on top of a SiO_2_ thin film with length *l*, width 2b, and two contact pads on each side. Heaters in this work had theoretical lengths from 9 to 15 mm and widths from 2 to 6 µm. The image is not depicted to scale.

The 3*ω* method, as used today, was first introduced by Cahill et al. in 1990 [28] and is widely used to measure thermal properties of bulk and thin film materials [29,30,31]. For the application of the 3*ω* method, a metal heater, as can be seen in Figure 1, has to be deposited on top of the sample. 

The 3*ω* method has a fast thermal response time, which allows for quick measurements; it is robust against convection and radiation losses; and it is insensitive against the boundary condition between substrate and environment [32]. To measure electrically conducting films, an electrically isolating layer between the heater and the film is needed [3]. 

A current with frequency 1*ω* flows through the heater, which is heated by joule heating as a consequence. The temperature and resistance of said heater change with frequency 2*ω*, and the voltage over the heater oscillates with frequency 3*ω*. This 3*ω* component contains information about the temperature amplitude of the heater, which is used to calculate the thermal conductivity of the material of interest [33]. However, since this 3*ω* component is quite small, a Wheatstone bridge and a lock-in amplifier (Anfatec eLock-In 204/2, Oelsnitz, Germany) are utilized to ensure reliable detection. The bridge circuit is illustrated in Figure 1. The measurement is performed for frequencies between 10 Hz and 10 kHz.

To heat the sample, an AC current with frequency *ω* is sent through the heater. This current *I*(*t*) can be expressed by [3]:

(1) $$I\left(t\right)=I_{0}\times \cos\left(\omega t\right),$$

where *I*_0_ is the current amplitude. The dissipated heating power *P*(*t*) can be written as:

(2) $$P\left(t\right)=I{\left(t\right)}^{2}\times R_{h}=\frac{I_{0}{}^{2}\times R_{h}}{2}\times \left(1+\cos\left(2\omega t\right)\right),$$

with *R_h_* as the electrical resistance of the heater. The dissipated power *P*(*t*) can be rewritten as [34]:

(3) $$P\left(t\right)=P_{DC}+P_{2\omega }\times \cos\left(2\omega t\right),$$

where *P_DC_* is the DC component and *P_2ω_*cos × (2*ωt*) is the AC component of the dissipated power. The power oscillates with frequency 2*ω*, and so does the temperature change over time of the heater ∆*T*(*t*). This can be written as [3]:

(4) $$\Delta T\left(t\right)=\Delta T_{2\omega }\times \cos\left(2\omega t+\varphi \right),$$

where ∆*T*_2*ω*_ is the temperature change amplitude, which contains the thermal conductivity information, and φ is the phase shift introduced because of the inertia of Joule heating [3]. As described by the temperature coefficient of resistance $\alpha_{R}$:

(5) $$\alpha_{R}=\frac{1}{R_{h,0}}\times \frac{dR_{h}\left(T\right)}{dT},$$

where *R_h_*_,0_ is the resistance of the heater at a reference temperature, the resistance of said heater changes with its temperature. Therefore, the resistance is time-dependent, which can be seen from Equation (4). This resistance change can be expressed as [3]:

(6) $$R_{h}\left(t\right)=R_{h,o}\times \left(1+\alpha_{R}\times \Delta T_{2\omega }\times \cos\left(2\omega t+\varphi \right)\right).$$

In this experiment, the voltage drop over the metal heater was measured. Therefore, an expression for this voltage had to be found, which was done by multiplying Equations (1) and (6):

(7) $$U\left(T\right)=I\left(t\right)\cdot R_{h}\left(t\right)$$

(8) $$U\left(t\right)=R_{h,0}\times I_{0}\times \cos\left(\omega t\right)+\frac{R_{h,0}\times I_{0}\times \alpha_{R}\times \Delta T_{2\omega }}{2}\times \cos\left(\omega t+\varphi \right)+\frac{R_{h,0}\times I_{0}\times \alpha_{R}\times \Delta T_{2\omega }}{2}\times \cos\left(3\omega t+\varphi \right)$$

(9) $$U\left(t\right)=U_{0}\times \cos\left(\omega t\right)+\frac{U_{0}\times \alpha_{R}\times \Delta T_{2\omega }}{2}\times \cos\left(\omega t+\varphi \right)+\frac{U_{0}\times \alpha_{R}\times \Delta T_{2\omega }}{2}\times \cos\left(3\omega t+\varphi \right),$$

information is contained in ∆*T*_2*ω*_, and therefore the 3*ω* component of *U*(*t*), is of great interest. Since the 3*ω* signal is a few magnitudes smaller than the 1*ω* signal [28,32], a lock-in amplifier (LIA) and a bridge circuit are used to detect the 3*ω* signal. From Equation (9), we receive:

(10) $$\Delta T_{2\omega }=\frac{2}{\alpha_{R}}\times \frac{U_{3\omega ,rms}}{U_{0,rms}}$$

with respect to the 3*ω* component only. Approximating the first harmonic voltage response as the input voltage signal, Equation (10) can be rewritten as:

(11) $$\Delta T_{2\omega }=\frac{2}{\alpha_{R}}\times \frac{U_{3\omega ,rms}}{U_{\omega ,rms}}.$$

The temperature change amplitude ∆*T*_2*ω*_ can be rewritten as:

(12) $$\Delta T_{2\omega }=\Delta T_{s}+\Delta T_{f}.$$

where ∆*T_s_* and ∆*T_f_* are the temperature change amplitudes of the bulk and the thin film. The LIA detects the bridge voltage *W*_3*ω*_. Therefore, the 3*ω* voltage drop over the heater has to be calculated by [25]:

(13) $$U_{3\omega ,rms}=\frac{R_{v}+R_{s}}{R_{v}}\times W_{3\omega }.$$

The temperature change amplitude of the bulk ∆*T_s_* can be calculated using [19]:

(14) $$\Delta T_{s}=\frac{P}{l\times \pi \times k_{s}}\times \left[\frac{1}{2}\times ln\left(\frac{k_{s}}{c\times p\times b^{2}}\right)+\eta -\frac{1}{2}\times ln\left(2\omega \right)\right],$$

where *η* was experimentally determined to be 1.05, according to Lee and Cahill [19], and c and *p* are the specific heat capacity and the density of the substrate, respectively.

To obtain the temperature change amplitude of the thin film ∆*T_f_*, only ∆*T_s_* has to be subtracted from ∆*T*_2*ω*_:

(15) $$\Delta T_{f}=\Delta T_{2\omega }-\Delta T_{s}.$$

The thermal conductivity of the thin film *k_f_* can finally be calculated by [3,27]:

(16) $$k_{f}=\frac{P\times d_{f}}{2b\times l\times \Delta T_{f}},$$

where *d_f_* is the thin film thickness. It should be noted that there are a variety of other ways of evaluating 3*ω* measurements. For more detailed information about other approaches, see [20,27,34,35,36].

### 2.2. Prerequisites for the 3-Omega Method

The following approximations are prerequisites for the theory explained above or were determined by simulations or experimental results [27,28,32,37,38,39]. By fulfilling all of those requirements, the valid heater geometries and measurement frequencies are defined, achieving a theoretical error of less than 1%. All approximations can be seen in Table 1, where $\lambda =\sqrt{D/\omega }$ is the thermal wavelength, D is the thermal diffusivity, h is the heat transfer coefficient for convection and radiation, and the indices x and z describe the anisotropy in the in-plane and cross-plane directions, respectively [27,28,32,37,38,39]. The last requirement shown in Table 1 can be mostly neglected, and if it is, the resulting error is still below 3% even for values > 4.8 [32].

### 2.3. Investigations on Heater Dimensions Using Laser Scanning Microscopy

In this work, a laser scanning microscope (LSM; Zeiss LSM 800, Oberkochen, Germany) was used to measure both the length and width of the heater with great precision. This was mandatory, as both parameters do have a significant influence on the result. The length of each heater was measured using an optical stitching method integrated into the LSMs’ ZEN-Software, enabling high-resolution images at large scales.

**Figure 2:**
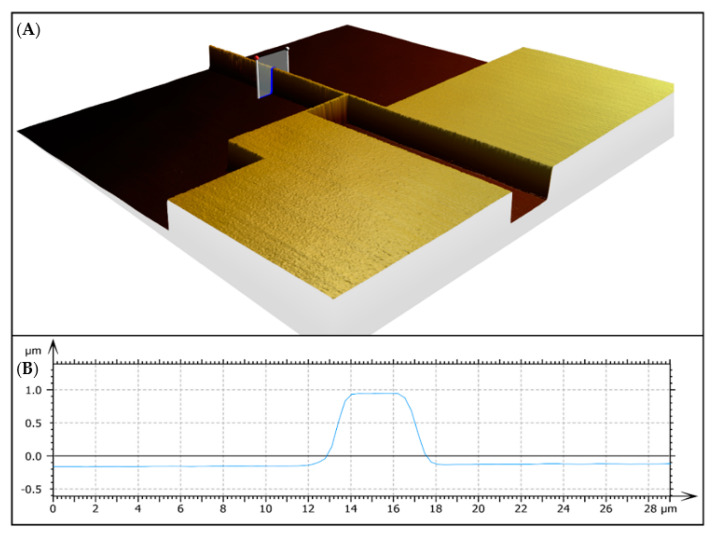
Z-scan performed with LSM: (**A**) 3D image created from z-scan showing the contact pads and the heater line itself. (**B**) Cross-section of the heater along the marker in (**A**).

Because of the deposition process, the heaters did not have perfect rectangular cross-sections, as can be seen in Figure 2. Thus, it was necessary to measure the width at the contact area, which is not possible with a normal optical microscope. Therefore, a z-scan using the built-in laser, featuring a wavelength of 405 nm, was performed, which delivered a high-resolution 3D image of the heater. An example is shown in Figure 2. The deviation from the theoretical deposited value to the real measured value was typically around 2 µm, which would lead to a thermal conductivity error of 20–50% depending on the heater width.

### 2.4. Materials

#### 2.4.1. Silicon Dioxide

Silicon dioxide (SiO_2_) thin films are widely used in micro- and nanoelectronics and semiconductor devices [40]. The main field of application for SiO_2_ is as a gate oxide in said semiconductor devices, for example, MOS structures. Because of its very well-known manufacturing methods, it is also used in a variety of other applications, such as separating individual chips from each other in integrated circuits or optical technologies [40]. In this work, SiO_2_ was taken as a reference, since its thermal conductivity for film thicknesses even below 100 nm is well known. These reference values are presented in Table 2. The samples used in this work were cut from eight-inch silicon wafers (GlobalWafers Co., Ltd., Hsinchu, Taiwan) with a thickness of 730 µm. They exhibited SiO_2_ thin films with thicknesses of 107 nm, 510 nm, and 1018 nm deposited on top using plasma-enhanced chemical vapor deposition (PECVD) with a deposition temperature of 400 °C.

#### 2.4.2. Aluminum Oxide

All Al_2_O_3_ thin films investigated in this work were manufactured using atomic layer deposition (ALD) (Infineon Technologies AG, Regensburg, Germany). Depending on the process temperature, the ALD Al_2_O_3_ thin film was usually amorphous after deposition [39,45,46]. Aarik et al. used different process temperatures ranging from 200 to 760 °C and found that crystalline films of 110 nm thickness could be manufactured using process temperatures exceeding 600 °C [45]. The finished sample could then be annealed, allowing the amorphous material to form crystals and become fully crystalline as long as the right parameters were chosen. Jakschik et al. investigated 3 to 8 nm thin films and found crystallization for temperatures above 900–1000 °C [47], while Zhang et al. found crystallization temperatures of 1050–1150 °C for a film thickness of 47 nm [39]. Additionally, temperatures over 1200 °C seem to destroy the crystalline structure [39]. It must be noted that Jakschik used 60–1800 s as process time, while Zhang used only 90 s [39,47]. With lower process temperature, longer process duration is needed for crystallization [47], while in general higher temperatures yield better crystallization results [39]. With high annealing temperatures, the density of the material increases, while the film thickness decreases [39,45,46,47]. Reported thickness losses from amorphous to crystalline material have ranged from 25.6 to 20.1 nm and from 47 to 40 nm [39,46].

**Figure 3:**
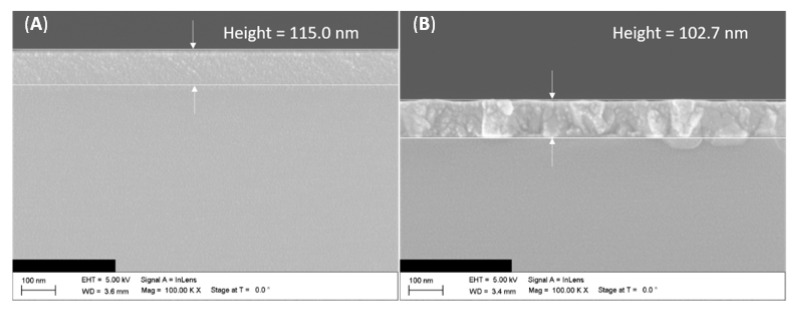
Scanning electron microscope (SEM) image of a cross-section of Al_2_O_3_ thin film samples (**A**) without annealing and (**B**) with annealing at 1000 °C.

The aluminum oxide films in this work were deposited using low-pressure atomic layer deposition (LP ALD) at a temperature of 350 °C. Ozone (O_3_) was used as an oxygen source, and trimethylaluminum (TMA) was used as an aluminum source. For our deposition temperature of 350 °C, the Al_2_O_3_ film was expected to be amorphous at first. Comparing the different annealing times and temperatures, it should be safe to assume that our Al_2_O_3_ film was crystalline, as it was annealed at 1000 °C for one hour. Moreover, the crystalline film should have had a lower thickness than the other sample. Scanning electron microscope (SEM) investigations of our samples confirmed the film thickness variations and the forming of crystalline structures, as can be seen in Figure 3. The thin film without annealing had a thickness of 115 nm which nearly matched the theoretical deposition target of 113 nm, while the thin film annealed at 1000 °C was only 102.7 nm thin, reflecting a decrease of 12.3 nm. The film was also clearly amorphous before annealing and showed some sort of crystallization afterward. The samples used in this work were cut from eight-inch silicon wafers with thicknesses of 730 µm and exhibited Al_2_O_3_ thin films of 115 nm and 102.7 nm thickness deposited on top using LP ALD. A list of all samples is depicted in Table 3.

### 2.5. Sample Structure

**Figure 4:**
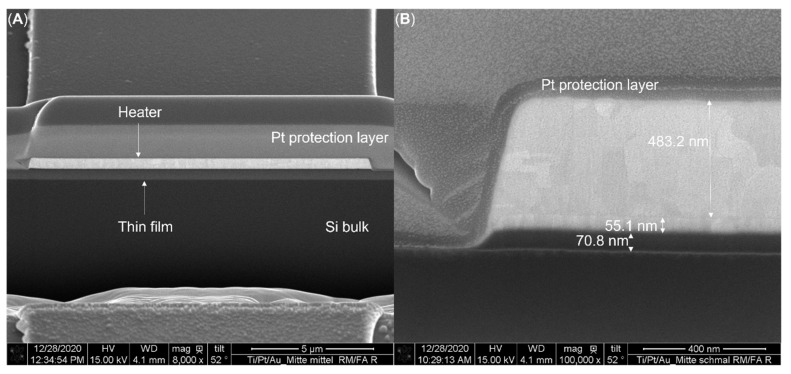
Cross-sectional SEM images of a heater structure (produced with FIB-cut). The Pt layer on top of the heater was used to protect the surface from damage due to the FIB process. It was deposited only on this specific sample and was not present on the devices that were measured. (**A**) shows a cross-section of a sample with substrate, thin film, and heater from bottom to top. (**B**) shows a cross-section of the heater, revealing the titanium (70.8 nm), platinum (55.1 nm), and gold (483.2 nm) stack.

All samples used in this work were based on the same layout. Each sample was 1 piece out of 42 pieces cut from a whole eight-inch wafer. Each wafer consisted of 730 µm-thick p-type Si bulk with a specific resistance of 9–18 Ω·cm with a thin film of SiO_2_ with a thickness of 107 nm, 510 nm, or 1018 nm on top. Alternatively, there was an Al_2_O_3_ thin film with a thickness of 115 nm or 102.7 nm on top, either unannealed or annealed at 1000 °C, respectively. In order to apply the 3*ω* method, a 620 nm thin titanium, platinum, and gold heater structure had to be deposited on top of the thin film. The main heater consisted of a 500 nm thin gold layer. A 120 nm thin titanium and platinum stack below was necessary to improve the adhesion between the gold and the surface. As illustrated in Figure 4, the titanium and platinum layers exhibited thicknesses of theoretically 60 nm each. Because of process inaccuracies, the actual heater height was around 610 nm.

**Figure 5:**
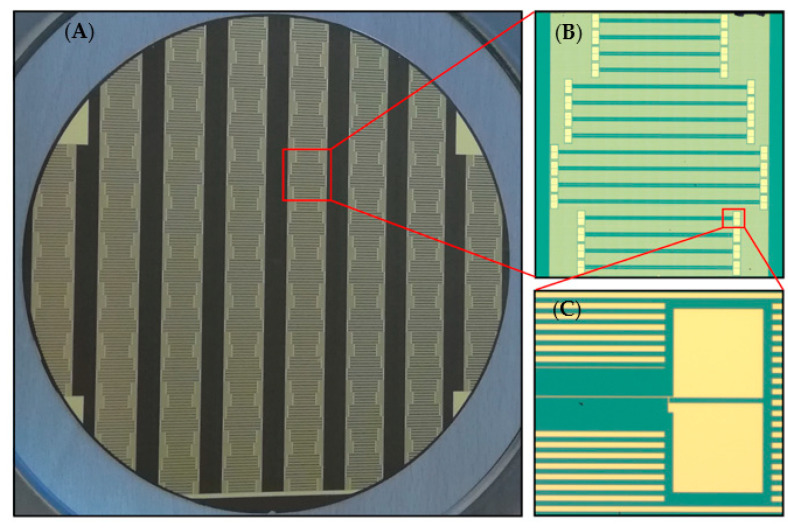
(**A**) Optical image of a complete wafer with repeating 3*ω* structures. (**B**) Optical microscope image of a full set of 16 heaters with different lengths and widths (zoomed into (**A**)). (**C**) Microscopic image of the contact pads on each side (zoomed into (**B**)).

Each heater consisted of a thin gold strip with two contact pads on each end. There were four adjacent heaters with the same length l and different widths 2b. This group of four heaters was repeated four times with different heater lengths l. The widths 2b ranged between 2 and 6 µm, and the lengths l ranged between 9 and 15 mm. In total, 16 different heaters were repeatedly arranged in a straight line over the whole wafer. The wafer held eight lines with 8.5 mm distances between them and widths of 16.3 mm. Each cut piece held two pairs of 16 heaters. The contact pads were 500 by 500 µm in size. A complete wafer is illustrated in Figure 5.

## 3. Results and Discussion

### 3.1. Measurement of Temperature Coefficient of Resistance

The temperature coefficient of resistance (TCR) $\alpha_{R}$ of the heater greatly influences the measurement result and therefore has to be determined very precisely. In most cases, it is not possible to use literature values, as the heater normally consists of more than one material. However, we noticed that variations between different heaters from one wafer were quite small, and thus, an average value could be used after measuring the TCR for a sufficient number of heaters. It is recommended to perform these measurements each time a new wafer, or in general a sample from another deposition process, is used.

**Figure 6:**
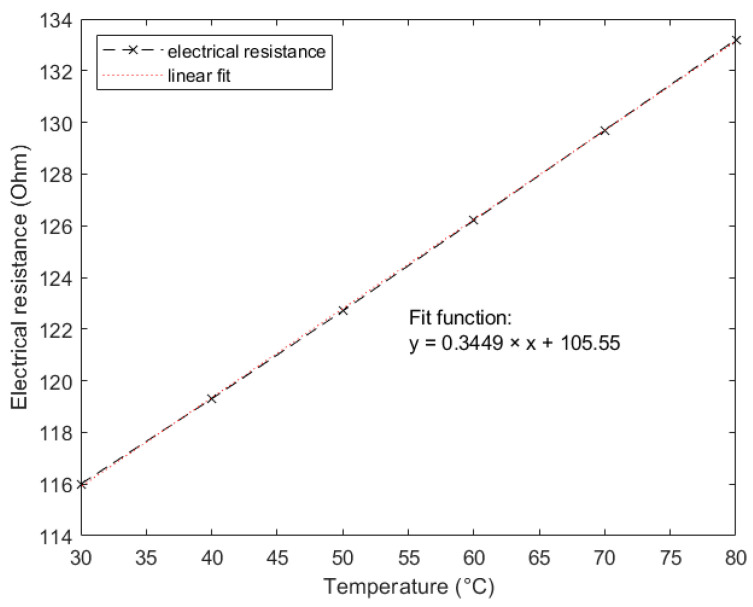
TCR measurement of 11 mm-long gold heater with 5 µm width on silicon dioxide thin film. Electrical resistance was measured at temperatures between 30 and 80 °C in steps of 10 °C. The resistance at measurement temperature *R_h_*_,0_ = 113.7 Ω.

To measure the TCR, the heater resistance was taken at measurement temperature, which was room temperature in most cases. Then, the sample was heated using a Peltier element, and the heater resistance was measured at different temperatures. The results for these measurements are displayed in Figure 6. The TCR was calculated using Equation (5).

### 3.2. Investigation of Possible Measurement Influences

Before any real measurements were carried out, investigation was attempted of the influence of as many potential sources of errors as possible. A summary of all measurements regarding this topic can be seen in Table 4.

**Figure 7:**
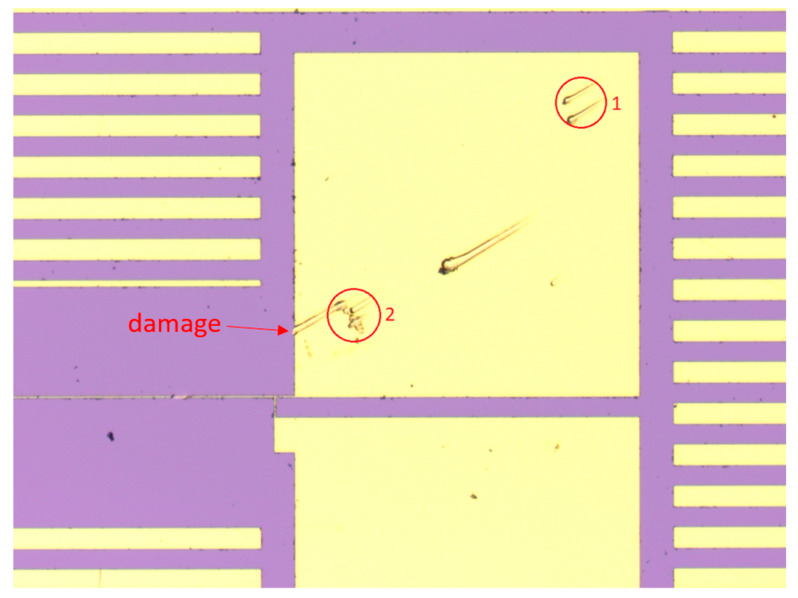
Contact pad of 3-Omega structure with two different contact positions. The positions were mirrored on the second contact pad. To the left of the second contact point, slight damage was induced to the contact pad after all other measurements were carried out.

**Figure 8:**
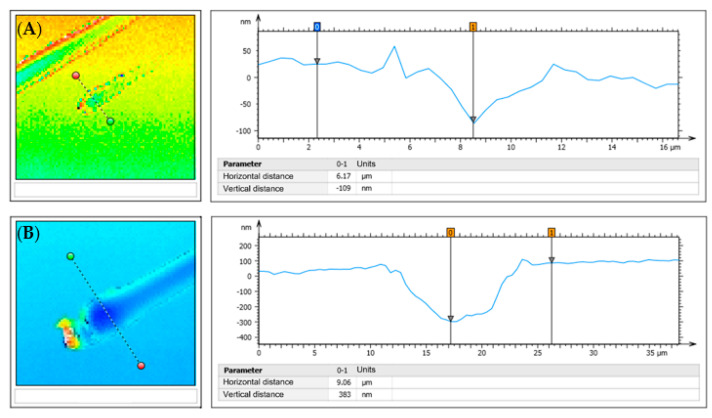
LSM measurements of penetration depths for (**A**) contact force 1 and (**B**) contact force 2. The left images show the complete LSM scans, while the right images show the cross-sections indicated in the left images.

- Bridge balancing frequency: The Wheatstone bridge was balanced before the measurement at one specific frequency, while the measurement itself took place at frequencies between 10 Hz and 10 kHz. Frequencies of interest were between 100 Hz and 1 kHz, as calculated from equations in Table 1. Therefore, the bridge balancing frequency was set within this range. Three measurements with different bridge balancing frequencies were taken.
- Contact position on the contact pads: The heater structures were connected to the measurement setup using contact needles. The contact positions were chosen in such a way that the distance to the heater itself was as large or as small as possible. A depiction thereof is shown in Figure 7.
- Contact force: It is possible to apply different contact forces to the needles. Unfortunately, there was no way to measure exact forces in this setup. Consequently, undefined small and high contact forces were specified through the penetration depth into the gold contact pad. This penetration depth was obtained using the LSM. The measurement results are illustrated in Figure 8. Contact force 1 corresponded to a penetration depth of 100 nm, and contact force 2 corresponded to a penetration depth of 380 nm.
- Measurement delay: This parameter defined how long the LIA stayed at one frequency to ensure a stable signal before executing the measurement. This was important because after approaching a new measurement frequency, the sample needs some time to adjust, as the thermal wave is dependent on the frequency and therefore changes after each frequency step. Two measurements were carried out; the delay time was set to 5 s for the first and to 20 s for the second measurement.
- Damaged pad structure: It should be obvious that damaged structures influence the measurement. However, already, minor damages, as shown in Figure 7 on the left side of the second contact point, did significantly influence the measurement and therefore could not be ignored.
- Native oxide layer: A native oxide layer forms on top of the substrate before any film can be deposited. To obtain the exact thickness of this layer, a pure substrate wafer was examined using a special ellipsometer, indicating a native oxide thickness of 1.03 nm. This finding was supported by Morita et al. [48]. Even if assuming a very low thermal conductivity of 0.15 Wm^−1^ K^−1^ for this oxide layer, the thermal resistance was 6.9 × 10^−9^ m^2^ KW^−1^, which was around two magnitudes lower than the total interface resistance of the samples (R_i_ = 2.6 × 10^−7^ m^2^ KW^−1^) and could therefore be neglected. It should be noted that the ellipsometer measurement result included airborne molecular contamination (AMC). AMC contributes typically to half of the measured thickness and is removed prior to thin film deposition by heating. Thus, the real native oxide thermal resistance was even lower. SiO_2_ and Al_2_O_3_ are both inert against oxidation in air or water, and therefore, no oxide could form on top of the deposited thin film.
- Influence of heater temperature on thin films: According to FEM simulations carried out in COMSOL Multiphysics^®^ (version 6, 2021, Comsol Multiphysics GmbH, Göttingen, Germany) the maximum DC temperature rise was 1.69 K, and the temperature amplitude was 1.78 K. Thus, the heater temperature rise was much lower than the deposition temperatures of 400 °C and 350 °C for SiO_2_ and Al_2_O_3_, respectively. Therefore, no change in thin film properties was expected.

As depicted in Table 4, the results showed that the heater structure had to be in flawless condition for stable and precise results, while all other parameters did not influence the measurement result significantly.

### 3.3. Silicon Dioxide

The material of interest in this section was SiO_2_, as for this material, plenty of thermal conductivity data for different film thicknesses are available, as can be seen in Table 2. With this data, the measurement results could be verified, and proper investigation of the heater geometry could be performed. Each measurement was repeated at least two times. Between measurements of the same geometry, at least one hour passed to guarantee that the heater and thin film below had sufficient time to cool down and return to their original states. 

**Figure 9:**
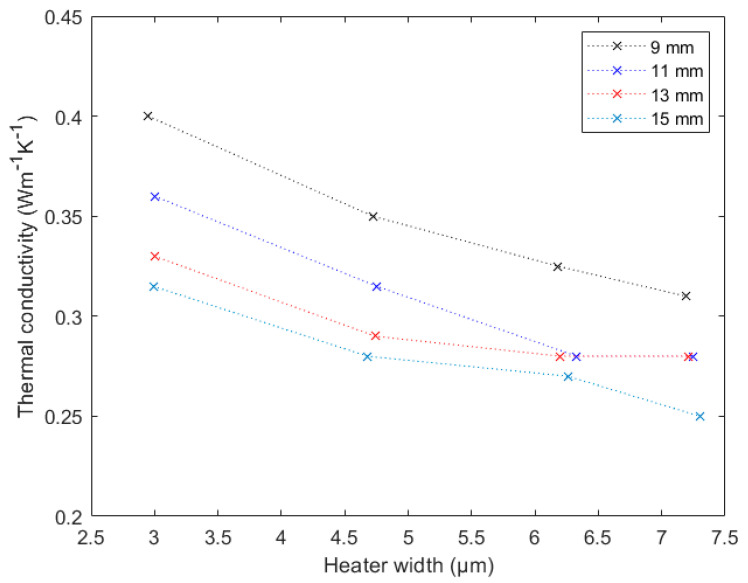
Measured thermal conductivity of 100 nm thin SiO_2_ film. The different colors correspond to different heater lengths as indicated in the inset.

First, the sample with a film thickness of 107 nm was investigated. The results can be seen in Figure 9. The approximations suggested that a greater heater width should lead to a more precise result, because the cross-plane thermal conductivity influence becomes dominant. Three of the four heaters, namely the 9 mm-, 11 mm-, and 13 mm-long heaters, showed exactly this behavior. The graphs flattened out for greater heater widths, and it seemed as if they approached a certain value. This stable value was already reached for the 11 mm- and 13 mm-long heaters. Furthermore, these two heaters approached the exact same thermal conductivity value, and therefore, these two lengths seemed to yield the most reliable results. The 9 mm-long heater could approach the same value as the wider heaters, while the 15 mm-long heater drifted away after initially approaching a similar value. The reason for the latter behavior is unknown, and more research has to be done to ascertain it.

**Figure 10:**
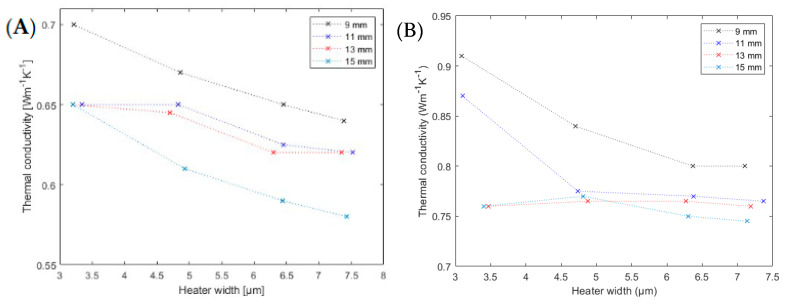
Measured thermal conductivity of (**A**) 500 nm and (**B**) 1000 nm thin SiO_2_ films. The different colors correspond to different heater lengths as indicated in the inset.

To verify these assumptions, the results for the 510 nm and 1018 nm thin films are illustrated in Figure 10. For those two samples, the requirements shown in Table 1 were not truly fulfilled, but the deviation was quite small, and the results should therefore still be reasonable. As both graphs showed similar behavior as stated before, this assumption seemed to be correct. The heaters with lengths 11 mm and 13 mm approached the same value for greater heater widths, while the 9 mm-long heater approached a slightly higher value. For the 510 nm thin film, the 15 mm-long heater also seemed to approach a certain value, but one considerably lower than our other values. The 11 mm- and 13 mm-long heaters delivered nearly the same values and thus supported our thesis from above. For the 1018 nm thin film, the 15 mm-long heater at first approached a similar value as the 11 mm- and 13 mm-long ones. This was the same as for the 107 nm thin film. Based on these measurements, we can state that both the heaters with lengths of 11 mm and 13 mm delivered correct results for a sufficiently wide heater.

**Figure 11:**
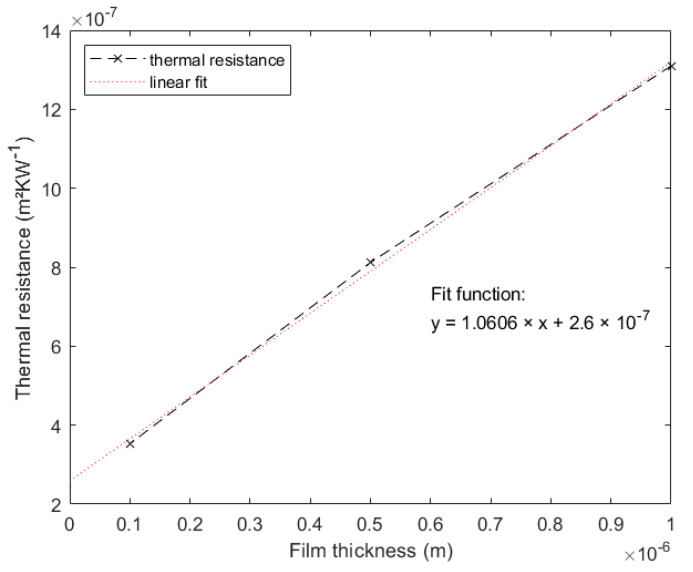
Thermal resistance of SiO_2_ thin film for different film thicknesses. A linear fit with the corresponding fit function is displayed, also.

Another way to verify these results is to take a look at the thermal resistance *R_f_* vs. the film thickness. *R_f_* is calculated using [20,25]:

(17) $$R_{f}=\frac{d_{f}}{k_{f}}=R_{i}+\frac{d_{f}}{k_{i}},$$

where *R_i_* is the total thermal interface resistance and *k_i_* is the intrinsic thermal conductivity. For the thermal conductivities of the films *k_f_*, the values for the 11 mm long heaters with a width of 6 µm were chosen. As stated by Yamane et al. [25] and Kim et al. [20], these two values should be linearly dependent for films of which the microstructure is independent of film thickness. This proves true for SiO_2_. If this was true for our results, it would attest the overall coherence of the previously described measurements. For our measurements, this graph is presented in Figure 11. There was clearly a strong linear dependency, which was underlined by a linear fit with *R*^2^ of 0.998. Looking at Equation (17), it becomes clear that it is possible to calculate the total thermal interface resistance and the intrinsic thermal conductivity with the help of Figure 11 and the linear fit equation, where *k_i_* = 1/slope = 0.94 Wm^−1^ K^−1^ is the slope and *R_i_* = 2.6 × 10^−7^ m^2^ KW^−1^ is the intersection with the *R_f_* axis.

According to the obtained findings, the thermal conductivity could be obtained by the investigation of either the 11 mm- or 13 mm-long heater wider than 6.5 µm. The results are shown in Table 5.

### 3.4. Aluminum Oxide

**Figure 12:**
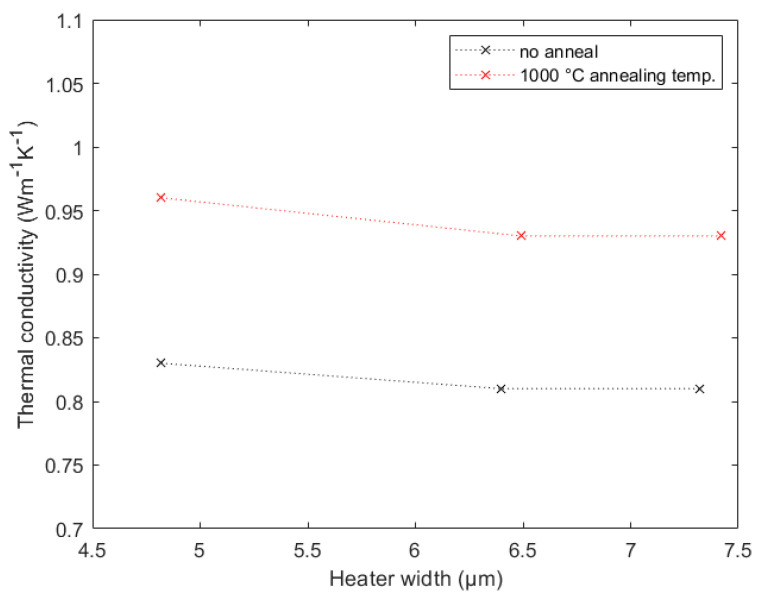
Measurement results for the 115 nm thin Al_2_O_3_ film and the 102.7 nm thin Al_2_O_3_ film with annealing at 1000 °C.

The Al_2_O_3_ samples with and without annealing at 1000 °C were investigated using the newfound promising heater structures with a length of 11 mm and widths between 5 and 7 µm. The results are shown in Figure 12.

As expected, both samples showed the desired trend and approached a stable value. The final thermal conductivity values can be seen in Table 6. As described in Section 2.4, after the annealing process, the Al_2_O_3_ film should no longer have been amorphous, since a certain type of crystallization was observed. Therefore, the thermal conductivity was expected to be higher, which was definitely the case. This thesis has been supported by numerous studies on different materials [49,50,51,52].

There are many different manufacturing processes for Al_2_O_3_ films, and therefore, it was quite hard to find good reference values. However, it was possible to find one reference for the sample without annealing that utilized exactly the same procedure as in the present work. Our result correlated with that from reference [53] very well, and therefore, the choice of the heater geometry was confirmed once more. To the best knowledge of the authors, there are no references for the annealed sample yet, as usually, the annealing times in the literature have been in the range of seconds or minutes, while we used an annealing time of 1 h.

## 4. Conclusions

A detailed inspection of the influence of various heater designs on the cross-plane thermal conductivity of different SiO_2_ and Al_2_O_3_ thin films was conducted. It was demonstrated within over 200 measurements that, even following all commonly used assumptions, the estimated thermal conductivity differs for various heater structures. It was revealed that heater structures of lengths between 11 and 13 mm with widths of 6.5 µm or more delivered reliable results for thin films with thermal conductivities *k_f_* < 1 Wm^−1^ K^−1^. To further evaluate the quality of the heater designs, tests on Al_2_O_3_ thin films were performed so that more reliable results could be obtained. It was demonstrated that the shown annealing process on Al_2_O_3_ thin films resulted in a reduction in film thickness and increased thermal conductivity. Furthermore, clear signs of crystallization of the Al_2_O_3_ films upon annealing were observed by SEM in the form of cone-shaped grains with diameters of up to 100 nm.

## Figures and Tables

**Table 1:** Requirements for choosing valid heater geometries and measurement frequencies.

Desired Approximation	Equation
Film heat flow is quasistatic, indicating that the material does not store energy and heat spreads equally [32,37]	$\frac{\lambda_{f}}{d_{f}}>5.7$
Substrate is semi-infinite [27,32]	$\frac{d_{s}}{\lambda_{s}}>2$
Heater counts as a line source [27,32]	$\frac{\lambda_{s}}{b}>5$
Heater counts as infinitely long [32]	$\frac{l}{\lambda_{s}}>2$
Heater is considered massless to neglect the volumetric heat capacity of the heater [27,32,37]	$\left(\frac{c_{h}\times p_{h}}{c_{f}\times p_{f}}\right)\left(\frac{d_{h}\times d_{f}}{\lambda_{f}{}^{2}}\right)<0.01$
Convection and radiation losses are negligible [28,32]	$max\left[\left(\frac{h\times d_{f}}{k_{f}}\right),\left(\frac{h\times \lambda_{s}}{2k_{s}}\right)\right]<0.01$
Substrate is isothermal [27,32]	${\left(\frac{k_{f}}{k_{s}}\right)}^{2}<0.01$
Heat flow through thin film is one dimensional [27,32]	(bdf)(kfzkfx)12>5.5
Heater thickness does not influence measurement [38]	$\frac{d_{h}}{2b}<0.05$
Influence of native oxide layer is neglectable [38,39]	$\frac{\pi \times k_{s}\times 2\times 10^{-9}}{2b\times k_{fz}\times ln\left(\frac{d_{s}}{b}\right)+1.0484}<0.01$
Influence of contact pads is neglectable [38]	$\frac{l}{2b}>600$
Heater counts as uniform heat source [32]	$\left(\frac{d_{h}\times d_{f}}{b^{2}}\right)\left(\frac{k_{h}}{k_{f}}\right)<1$

**Table 2:** Literature values of the thermal conductivity of silicon dioxide for different film thicknesses. The last 3 rows sum up all shown references.

Film Thickness (nm)	Thermal Conductivity (Wm^−1^ K^−1^)	Reference
100	0.05–0.08	Griffin, 1994 [41]
100	0.73–0.9	Lee, 1997 [19]
200	0.85–1.15	Goodson, 1993 [42]
500	0.2–0.3	Griffin, 1994 [41]
500	0.59–0.77	Govorkov, 1997 [43]
500	1.15–1.4	Goodson, 1993 [42]
1000	0.35–0.6	Griffin, 1994 [41]
1000	0.59–0.61	Govorkov, 1997 [43]
1000	0.1–1.3	Cahill, 1994 [44]
100	0.05–0.9	-
500	0.2–1.4	-
1000	0.35–1.3	-

**Table 3:** Overview of all sample materials, deposition methods, film thicknesses, and annealing temperatures.

Bulk Material	Film Material	Deposition Method	Film Thickness (nm)	Annealing Temperature (°C)
Si	SiO_2_	PECVD	107	—
Si	SiO_2_	PECVD	510	—
Si	SiO_2_	PECVD	1018	—
Si	Al_2_O_3_	LP ALD	115	—
Si	Al_2_O_3_	LP ALD	102.7	1000

**Table 4:** Results for different potential measurement influences for a 1 µm thin SiO_2_ thin film. The deployed heater geometry, investigated parameters, and measurement results are shown.

Heater Length (mm)	Heater Width (µm)	Balancing Frequency (Hz)	Thermal Conductivity (Wm^−1^ K^−1^)
11.086	3.10	100	0.84
11.086	3.10	500	0.83
11.086	3.10	1000	0.83
Contact position			
13.096	3.47	1	0.72
13.096	3.47	2	0.72
Contact force			
13.098	4.88	1	0.73
13.098	4.88	2	0.73
Measurement delay			
11.092	6.38	5 s	0.77
11.092	6.38	20 s	0.77
Pad condition			
13.096	3.47	intact	0.72
13.096	3.47	damaged	0.68

**Table 5:** Final measurement results for different SiO_2_ thin films.

Film Thickness (nm)	Thermal Conductivity (Wm^−1^ K^−1^)	Reference Values (Wm^−1^ K^−1^)
107	0.28	0.05–0.9 [19,41]
510	0.62	0.2–1.4 [41,42,43]
1018	0.77	0.35–1.3 [41,43,44]

**Table 6:** Final thermal conductivity measurement results for the different Al_2_O_3_ samples.

Annealing Temperature (°C)	Film Thickness (nm)	Thermal Conductivity (Wm^−1^ K^−1^)	Reference Value (Wm^−1^ K^−1^)
-	115	0.81	0.8 [53]
1000	102.7	0.93	-

## Data Availability

The data presented in this study are available on request from the corresponding author.
